# Psychological safety between pediatric residents and nurses and the impact of an interdisciplinary simulation curriculum

**DOI:** 10.1186/s12909-022-03709-9

**Published:** 2022-08-29

**Authors:** Courtney Haviland, Janet Green, Kristina Dzara, Wendy O. Hardiman, Emil R. Petrusa, Yoon Soo Park, Ariel S. Frey-Vogel

**Affiliations:** 1 Department of Pediatric Emergency Medicine, Hasbro Children’s Hospital, 593 Eddy St., Providence, RI 02903 USA; 2 Department of Nursing, Mass General for Children, 55 Fruit St., Ellison 17, Boston, MA 02114 USA; 3 Department of Biomedical Informatics and Medical Education and Center for Leadership and Innovation in Medical Education, University of Washington School of Medicine, 1959 NE Pacific St., Seattle, WA 98195 USA; 4 Mass General for Children, Department of Nursing, 55 Fruit St., Ellison 17, Boston, MA 02114 USA; 5 Department of Surgery, Harvard Medical School and Massachusetts General Hospital, Bartlett Hall Second Floor, 55 Fruit St., Boston, MA 02114 USA; 6 Department of Emergency Medicine, Harvard Medical School and Massachusetts General Hospital, Bartlett Hall Second Floor, 55 Fruit St., Boston, MA 02114 USA; 7 Harvard Medical School and Mass General for Children, Department of Pediatrics, 175 Cambridge St, Fifth Floor, Room 587, Boston, MA 02114 USA

**Keywords:** Psychological safety, Interprofessional simulation, Nurse/physician relationship, Interprofessional teamwork

## Abstract

**Background:**

Effective teamwork in interdisciplinary healthcare teams is necessary for patient safety. Psychological safety is a key component of effective teamwork. The baseline psychological safety on pediatric inpatient healthcare teams is unknown. The purpose of this study is to determine the baseline psychological safety between pediatric nurses and residents and examine the impact of an interdisciplinary nighttime simulation curriculum.

**Methods:**

A convergent, multistage mixed methods approach was used. An interprofessional simulation curriculum was implemented fall 2020 to spring 2021. Qualitative focus group data and quantitative survey data on team psychological safety were collected and compared, both pre- and post-intervention and across nurses and residents. Thematic analysis of the qualitative data was conducted, and themes integrated with survey findings.

**Results:**

Data were collected from 30 nurses and 37 residents pre-intervention and 32 and 38 post-intervention, respectively. Residents and nurses negatively rated psychological safety (pre-intervention mean = 3.40 [SD = 0.72]; post-intervention mean = 3.35 [SD = 0.81]). At both times psychological safety was rated significantly lower for residents (pre-intervention mean = 3.11 [SD = 0.76], post-intervention mean = 2.98 [SD = 0.84]) than nurses (pre-intervention mean = 3.76 [SD = 0.45], post-intervention mean = 3.79 [SD = 0.50]), all *P* < .001. Qualitative analysis identified six integrated themes: (1) influence of existing relationships on future interactions, (2) unsatisfactory manner and frequency of communication, (3) unsatisfactory resolution of disagreements (4) overwhelming resident workload impairs collaboration, (5) interpersonal disrespect disrupts teamwork, and (6) interprofessional simulation was useful but not sufficient for culture improvement.

**Conclusion:**

Resident-nurse team psychological safety ratings were not positive. While interprofessional simulation curriculum shows promise, additional efforts are needed to improve psychological safety among residents and nurses.

## Background

Teamwork between nurses and physicians is both vital to patient care and difficult to do well which can lead to medical errors [1]. The Accreditation Council for Graduate Medical Education, in their Clinical Learning Environment Review Pathways to Excellence, has named ‘teaming’ as one of its core pathways, emphasizing its importance [2]. In academic medical centers, teamwork challenges are compounded by frequently changing teams comprised of people with varying levels of experience. Healthcare delivery is high stakes and sometimes high stress, which can challenge interpersonal interactions. Interpersonal dynamics can stress healthcare workers and contribute to feelings of burnout [3]. At our institution, an intervention which partners nurses with new interns starting at intern orientation has been implemented to improve identified nurse-resident relationship difficulties [4] which was found to expedite the formation of positive nurse-resident relationships [5].

Evidence has identified successful team attributes: psychological safety, member dependability, role structure and clarity, meaning of work, and impact of work. Psychological safety—by far the most important [6, 7]—is a shared belief that the team is safe for interpersonal risk taking, including asking for help, admitting errors, and seeking feedback without fear of retribution [8]. Teams with high psychological safety have more trust. Members of teams with high psychological safety are more willing to demonstrate vulnerability because they believe that rather than being judged, they will be recognized as working towards a team goal. They also feel empowered to disagree, without being disagreeable [8]. This is particularly important in health care, where acknowledging all members’ opinions can be the difference between making and preventing a medical error. Prior studies of healthcare teams have correlated high team psychological safety and effective disagreement management with improved patient outcomes [9–11].

This mixed methods study explored the existing resident-nurse team dynamics on an inpatient pediatrics floor through the lens of psychological safety and evaluated the impact of an interprofessional simulation curriculum on measures of psychological safety [8], quality of nurse-physician relationships [11], and quality of disagreement management [11].

## Methods

The study utilized a convergent, multistage mixed methods approach. Prior to interprofessional simulation curriculum implementation, qualitative data were collected via focus groups and quantitative data by survey. The curriculum (described below) ran for six months, fall 2020—spring 2021, after which the focus groups and survey were repeated.

### Setting and participants

Study participants were recruited from the nurses (*n* = 75) and pediatric residents (*n* = 65) who staff the inpatient pediatric floors of an urban pediatric hospital within a large academic medical center in the Northeast United States.

### Study recruitment

Participants were recruited using a convenience sample (email and paper advertisements). Pre-intervention focus groups were recruited from the nurses and residents who work on the inpatient floors. Post-intervention focus groups were recruited from the residents and nurses who attended at least one interprofessional simulation session. All nurses and residents who work on the inpatient floors were eligible to complete both the pre- and post-intervention surveys regardless of simulation session attendance.

### Data collection

The survey included three measures with existing validity evidence [9]. Section one is the Psychological Safety Scale [6], sections two and three are from the ICU Nurse Physician Questionnaire (ICUNPQ) [7] and respectively measure the quality of nurse-physician relationships and quality of disagreement management. All study team members reviewed the survey for content and three recent residency graduates pilot tested the survey for usability. Survey data were collected anonymously via RedCap.

A semi-structured interview guide for residents and nurses was created to represent survey constructs and refined by all study authors. The post-intervention interview guide was revised based on pre-intervention findings. The focus groups were conducted over video conferencing by a study team member (KD) who is an experienced focus group facilitator and does not supervise any participants. Nurses and residents participated in separate one-hour focus groups and were given a $15 gift card. The focus groups were audio recorded, professionally transcribed, and then reviewed and deidentified by KD.

### Interprofessional simulation curriculum

The medical team, composed of residents and nurses, participated in one-hour high fidelity manikin simulation sessions during the night shift once every 2–4 weeks. All sessions were conducted at night given the relative decrease in overall floor activity compared to during the day in order to allow for full participation. Seven sessions were completed in total over the September 2020 – March 2021 time period; the goal had been to have one every other week but they ended up occurring once a month due to difficulties with scheduling, holidays, and the patient care needs on the hospital floors. For each session, all of the residents working at night (2 PGY1, 1 PGY2, and 1 PGY3 or PGY4) were asked to participate; one resident would step away if they were required for patient care needs. Two of ten nurses working each night were required to attend. Sessions began with brief didactics highlighting the importance and inherent challenges of optimal team performance and the importance of and behaviors to improve team psychological safety. The teams then participated in a simulated medical scenario, after which the facilitators (CH, JG, WH) led a debrief of their interprofessional performance.

### Data analysis

Participant responses to items from each section were averaged to create composite psychological safety, quality of nurse-physician relationships, and quality of disagreement management ratings from each participant. An average Likert score of 4 or 5 (“agree” or “strongly agree”) for each survey section was considered a “positive” rating. Scores less than 4 were considered “not positive.” Descriptive statistics were performed. T-tests were used to compare pre- and post- intervention scores for each group and between groups. Effect size of the intervention was determined by Cohen’s *d*. Data analysis was performed using IBM SPSS 24.

A thematic analysis of the focus group transcripts was conducted following the “five stages to qualitative research” framework [12]. There were three coders: two physicians (AFV and CH) and one nurse (JG). The coders independently read the first third of the nursing transcript and created preliminary codes. The coders compared, refined, and collapsed initial codes to start the codebook, then each read the rest of the first transcript, applied existing codes, and added additional codes. The coders again compared and collapsed codes and refined the codebook. The coders did the same for the resident transcript, adding additional codes as necessary. They finalized the pre-intervention codebook, ensuring triangulation between coders. The coders discussed recurring data patterns and combined codes into categories, then themes. Survey results and emerging themes were reviewed, integrated, and verified with the study team.

The codebook was similarly applied to the post-intervention focus groups, comparing pre- and post-intervention data, adjusting the categories and themes as needed, and integrating with post-intervention survey findings. One nurse and one resident focus group participant served as member checkers by critically reviewing the manuscript and providing feedback that was incorporated into the final version.

This study was approved by the Mass General Brigham Institutional Review Board.

## Results

There were seven total simulation sessions over a period of seven months. Table 1 indicates nurse or resident engagement in the simulation sessions, surveys, and focus groups. While only 35% of residents overall participated in the simulation curriculum, only 39 residents worked at night on the hospital floors during the study period and so 59% of the potential residents who could have participated did. Similarly, while 19% of all nurses participated in the simulation curriculum, only 35 worked at night during the study period and so 43% of potential nurses who could have participated did. All post-intervention focus group participants participated in the simulation curriculum.

**Table 1:** Participant engagement in study activities

Activity	Nurses N (%)	Nurse Number of Years Worked (ave + /_ SD)	Residents N (%)	PGY year (PGY year: # participants
Pre-intervention survey	30 (40%)	14.2 ± 10.3	37 (57%)	PGY1:13 PGY2:15 PGY3: 8 PGY4:1
Pre-intervention focus group	4	17.8 ± 11.4	4	PGY1:1 PGY2:3 PGY3:0 PGY4:0
Simulation Curriculum	14 (19%)		23 (35%)	
Post-intervention survey	32 (43%)	12.1 ± 6.6	38 (58%)	PGY1:14 PGY2:13 PGY3:11 PGY4: 0
Post-intervention focus group	3	Not recorded	5	Not recorded

Our analysis resulted in six integrated themes. Five were identified as impacting psychological safety both pre-and post-intervention: (1) influence of existing relationships on future interactions, (2) unsatisfactory manner and frequency of communication, (3) unsatisfactory resolution of disagreements (4) overwhelming resident workload impairs collaboration, (5) interpersonal disrespect disrupts teamwork. Post-intervention, we found (6) interprofessional simulations useful but not sufficient for culture improvement.

### Theme 1: Influence of existing relationships on future interactions

Respondents consistently reported that existing relationships and prior interactions impact their work together. This applied both between individuals and groups.

“…[I]t’s not just…two people who don’t have a preexisting relationship going about the business of the floor work. There is significant background personal relationship in every interaction.” (Resident, pre-intervention [pre])

Residents, in particular, described predicting how a clinical encounter would go based on the nurse involved.

“There’s an element of knowing when an admission may be…more challenging or less challenging depending on…the…level of conflict that they often bring…” (Resident, pre)

While specific personal relationships mattered, psychological safety was also impacted by the overall relationships between groups. Both groups struggled to balance the resident as team leader with the more experienced nurse as patient advocate.

“I want to totally respect the decades of experience in some cases of the nursing team, but at the same time, there are things that happen because a physician orders them.” (Resident, pre)

“The bottom line is I will do what is necessary for the patient, but if it really doesn’t sit well with me and I want to talk about why we’re going to do what we’re going to do, and they’re like, ‘Why won’t you just do it?’ I'm not going to just do it.” (Nurse, pre)

Quantitative data reinforced these sentiments. The aggregate means from nurses and residents showed low psychological safety, with no significant change in rating between interventions (pre-intervention mean = 3.40, SD = 0.72; post-intervention mean = 3.35, SD = 0.81), *P* = 0.73. Residents rated psychological safety significantly lower than nurses both pre-intervention (resident mean = 3.11, nurse mean = 3.76*, p* < 0.001; Cohen’s *d* = 1.71) and post-intervention (resident mean = 2.98, nurse mean = 3.79; Cohen’s *d* = 1.93), *P* < 0.001. (See Table 2).

**Table 2:** Mean responses to survey subsections comparing role groups horizontally and pre- versus post-intervention vertically

Psychological Safety					
	Resident mean	SD	Nurse mean	SD	p
Pre	3.11	0.76	3.76	0.45	0.001
Post	2.98	0.84	3.79	0.50	0.001
p	0.49		0.85		
Relationship quality					
	Resident mean	SD	Nurse mean	SD	p
Pre	3.30	0.68	3.32	0.52	0.85
Post	3.14	0.77	3.34	0.56	0.23
p	0.35		0.92		
Disagreement management process					
	Resident mean	SD	Nurse mean	SD	p
Pre	3.17	0.61	3.63	0.71	0.006
Post	3.10	0.64	3.51	0.57	0.004
p	0.62		0.46		

Lack of pediatric resident autonomy was an additional contributor to this dynamic, especially when nurses would go to the resident’s supervisor upon disagreement.

“…[T]hen you end up in a situation where the senior resident and the attending aren’t really taking what the intern says seriously. So of course, the pediatric nurses also will wait to hear from the senior resident or the attending before they act on the plan.” (Resident, pre)

Both groups indicated that they valued the skills of the other, however neither group always felt valued.

“There are some that…do think I can actually take care of patients, and then there are those who just treat us like we know nothing.” (Resident, post)

“[W]e can say, ‘Oh, I’ve taken care of 50 of these kids, and…this is kind of the trajectory of what it looks like, so you might want to look into this.’ And when they take that in, I feel respected.” (Nurse, pre)

Nurses felt that relationships were more positive than residents, yet quality of relationship ratings were not positive for either group pre-intervention (resident mean = 3.30 [SD = 0.68], nurse mean = 3.32 [SD = 0.52]) or post-intervention (resident mean = 3.14 [SD = 0.77], nurse mean = 3.34 [SD = 0.56]). Comparisons of nurses and residents were not significant pre-intervention (*p* = 0.85) or post-intervention (*p* = 0.23) (Table 2).

### Theme 2: Unsatisfactory methods and frequency of communication

Both groups agreed that ideal team communication would involve open, transparent, regular, face-to-face interactions with all team members present.

“I think it works well when they come up to you in the morning and say, ‘Hey, I have this patient and that patient.…is there anything emergent right now or something on your mind?” (Nurse, pre)

Challenges arose when some team members were not included on rounds or when making plans or when regular check-ins did not occur.

“…Sometimes I feel like I won’t even know who’s covering my kid. They’ll round without me. They’ll put in orders without telling me. And to me, that's really frustrating.” (Nursing, post)

The goal for both groups was that all team members would be able to offer input and feel heard. Both groups were frustrated when they could not share.

“I feel like they were doing a disservice to the patient. It was more so - I wasn’t trying to fill my ego and say, ‘I think I’m right.’…I just felt more frustrated that I felt like they could’ve done more for the patient. ” (Nurse, post)

“I can just feel this sense of judgment. And I don’t think it’s just perceived. I know it’s there. And that’s such a barrier to having an authentic conversation.” (Resident, post)

Residents desired, but did not always receive, nursing input.

“This happens all the time where I have a sense that the nurse coming to me has an opinion on what to do about it, but then when I try to elicit what that is, then for some reason, they won’t tell me.” (Resident, pre)

Residents noted that their confidence level had an impact on communication success.

“…[A]s I have progressed in residency and I am more confident, then my communication style is different and more confident as well. And I don’t really have as many issues anymore.” (Resident, pre)

### Theme 3: Unsatisfactory resolution of disagreements

Participants revealed dissatisfaction with disagreement management processes. Neither group’s mean ratings were positive either pre-intervention or post-intervention where resident means were 3.17 (SD = 0.61) and 3.10 (SD = 0.64) and nurse means were 3.63 (SD = 0.71) and 3.51 (SD = 0.57), respectively. There was no difference between pre-intervention and post-intervention ratings within groups. Resident ratings were significantly lower than nurses’ at pre-intervention (Cohen’s *d* = 1.49) and post-intervention (Cohen’s d = 1.27) with *p* < 0.007 for both comparisons (Table 2).

Indeed, both groups felt their input was dismissed and that interactions were overly confrontational.

“… I think my most unsettling kind of disagreements are when I feel like…they’re not listening to me. I’m like, ‘There’s something wrong with this patient. We need to do something else.’” (Nurse, post)

The primary driver of disagreement was nurses’ belief that they better understood the impact of plans on patients, and resident desire of be respected as the care leader.

“… [W]e’re kind of pushing back because on the other end of that booklet of papers is a baby or a child who is the one getting woken up so that we just have numbers that aren’t going to change anything before they wake up in the morning.” (Nurse, pre)

“And even if I’m an intern and it’s my first week, ultimately, I have to put in the order, and it’s my name that gets associated with that order and that decision that has to be made by a physician.” (Resident, pre)

While both groups agreed that they would absolutely speak up in disagreement in matters of patient safety or high clinical importance, smaller disagreements were often avoided to prevent confrontation. These unresolved disagreements festered and impacted future interactions.

“But I never really got to be like, ‘How come we didn’t work this out?...’ I don’t think there’s closure to the disagreements…” (Nurse, pre)

“There’s some times where it’s as if nothing ever happened…And then, you’re just kind of left wondering at what point… that switch will happen again, and kind of being on guard in that way. And that’s also exhausting.” (Resident, pre)

### Theme 4: The overwhelming resident workload impairs collaboration

Both groups highlighted residents had an overwhelming workload. This frustrated their ability to collaborate with nurses and sometimes misaligned priorities:

“…[C]ommunication is poor because [the residents] can’t take the time sometimes to come speak to the nurse. They literally are running full speed ahead.” (Nurse, post)

Residents were emotionally taxed by negative interactions. This made them want to “give up” and further entrenched existing problematic relationship dynamics.

“And so it becomes a vicious cycle of nursing wants this, so residents do this so nursing feels like that was the right call…And so then, nurses, I think, have a falsely elevated sense of clinical evaluation that’s only true because I don’t want to die on every single hill that they make for themselves.” (Resident, post)

### Theme 5: Interpersonal disrespect disrupts teamwork

Both residents and nurses described rare, but high impact, episodes of interpersonal disrespect that made it difficult to work together. The groups felt disrespected in different ways. Nurses described being dismissed or unfairly blamed.

“You tell me ‘we hear you’re concerned,’ six times a shift, but nothing has changed towards the care of this patient.” (Nurse, pre)

Residents experienced aggressive behavior and yelling. They described a double standard for workplace expectations, believing they would face serious disciplinary action for behaviors they experienced frequently.

“… I was shouted at every single time that I walked by the nurse’s station… I was verbally assaulted for a good 48 hours.” (Resident, pre)

Both groups were affected by these episodes, but residents were more likely to feel that the negative experiences affected the overall relationship whereas nurses did not.

“I’ll be honest, as many good nurses as there are, it’s the negative actions from the not-so-good nurses that…ends up trumping the good relationship we have with good nurses.” (Resident, post)

“In each group [there’s] maybe a bad apple…. But I think in general…you can just go up to them and kind of speak about your patients and advocate for them and feel comfortable doing so.” (Nurse, post)

The emotional impact of these episodes weighed heavier on residents than on nurses. This was compounded by perceived lack of support by superiors.

“And leadership have mentioned to us things like, ‘Oh, yeah, they’ve always been hard to work with,’…as if that makes us feel better. But in fact, it makes us even more belittled and dismissed as if this is an issue that’s been longstanding, nobody cares.” (Resident, pre)

### Theme 6: Interprofessional simulation is useful but not sufficient for culture improvement

Interprofessional simulation was viewed positively by focus group participants and by survey ratings of perceived value (mean = 4.6) and enjoyment (mean = 4.7) Participants appreciated the in-situ aspect, since it offered a unique chance to practice medical responses with each other.

“Usually, our sims are – we don’t have actual nurses, it’s our attendings or chiefs pretending to be nurses. So, I thought it was great that we were actually able to work with nurses that we work with all the time but in a simulated environment.” (Resident, post)

They appreciated that it built familiarity between team members and offered a chance to practice “speaking up” in a safe space.

“Having the personal relationships with the residents… doing the simulation in a non-emergency situation, it helps you feel …more comfortable being like, ‘Maybe we should try this first,’ and just making sure that it’s a decision made as a team.” (Nurse, post)

However, neither group felt the lessons learned through the simulation curriculum were likely to improve the broader culture of psychological safety. There was a small Cohen’s *d* effect size for respondents who attended at least one simulation curriculum session (Cohen’s *d* = 0.2) which suggests that the simulation may have had a small positive influence on participants. Regardless, they believed the curriculum reinforced existing relationships.

“I think the same nurses who make psychological safety difficult to attain on the floors are the same nurses who make it difficult to attain in sim. And I don’t think that simulation changes that.” (Resident, post)

## Discussion

We sought to understand the baseline degree of psychological safety between nurses and residents on our pediatric inpatient floors and whether that changed upon interprofessional simulation curriculum implementation.

Our interprofessional simulation curriculum was designed to improve psychological safety between residents and nurses. While it was rated as valuable and enjoyable, there was only a small effect size for increased psychological safety ratings post-intervention. Our findings outline multiple challenges to building psychologically safe nurse-resident teams which were unaffected by the interprofessional simulation. The first is role tension. Residents are usually less experienced than nurses but expected to drive patient care, which often leads to a cycle of nurses not trusting residents, and residents feeling invalidated and not incorporating nursing concerns into their plans. This cycle continues even as resident experience and competence increases due to the prior breach in trust. The second hurdle is interaction dread. After a negative interaction, nurses and residents tend to avoid direct interactions the next time a problem arises, exacerbating the issue. The third hurdle is resident workload. Residents are overwhelmed by tasks, so effective and timely communication with nurses does not always happen. Nurses also cannot always be present on rounds when care decisions are made. These factors set up another cycle of neither group feeling heard or psychologically safe. The last major hurdle is the rare but egregious episodes of unprofessional behavior. Residents report that nurses yell at and belittle them while nurses report that residents create a façade of listening while actually ignoring them.

Overall, our findings reveal negative and even egregious interactions occur between nurses and residents and those negative interactions impact team members and their relationships. Descriptions of abusive interpersonal interactions in medicine are not new [13–15]. Negative interactions result in disengagement, burn out, and feelings of low self-worth [3]. Team members cannot feel psychologically safe when they perceive they are at risk of being yelled at and demeaned, or when they do not feel their contributions are valued by the other team members [8]. Improving the relationships between healthcare providers requires offering a safe space [16] to practice “positive” interactions and disagreement resolutions, encouraging baseline high regard between team members [16], building interprofessional familiarity, and modeling interpersonal feedback strategies [17]. The interprofessional simulation curriculum has potential, but improving psychological safety requires a culture improvement on the pediatric floors, and culture improvement requires more than a single intervention [18] and would require all nurses and residents (and likely the other healthcare providers working on the pediatric floors) to participate in any intervention introduced to improve psychological safety. Furthermore, simulation would likely need to be repeated longitudinally to have a positive impact on culture.

Our study has limitations. There were a small number of participants from a single institution. Because our surveys were anonymous, we could not precisely match up individuals who responded to pre- and post-intervention surveys. People who chose to respond to the survey may have been participants in the simulation who had a more positive perception of the effects of the simulation and it is possible we do not have a representative sample of respondents. Focus group participants may be intrinsically motivated to discuss this topic. Only one focus group for each role group was held at each time point and we cannot be certain that our themes fully capture the scope of resident-nurse team dynamics. Further, we do not know the level of experience of the nurses or residents who gave the specific comments quoted. The intervention pilot was short, and less than 50% of subjects participated; only nurses who work nights were exposed.

We plan to continue and expand the interprofessional simulation curriculum as we believe it is part of the solution to improving psychological safety, and that more sessions reaching more clinicians might increase that effect. However, systemic improvement is necessary to improve psychological safety. The pediatric general floor leadership in our institution is currently determining how to enact such culture improvement. It could be driven by interprofessional education about balancing the resident as leader and as learner, by decreasing resident workload to allow time for collaboration [19], and by upholding standards of professional behavior for all clinicians at the personal [20, 21] and departmental level.

## Conclusions

Resident-nurse psychological safety remains crucial for high-quality patient care. While our interprofessional simulation curriculum shows positive potential in mitigating psychological safety levels in the resident-nurse workforce, additional holistic solutions are likely needed to demonstrate sustained improvement. At our institution, resident-nurse team psychological safety ratings were not positive, and role tension, interaction dread, high resident workload, and episodes of unprofessional behavior were identified as major barriers. The amount of simulation training needed in our institution to help improve psychological safety between residents and nurses is yet to be determined.

## Data Availability

The datasets used and/or analyzed during the current study are available from the corresponding author on reasonable request.

## Abbreviations

ICUNPQ: Intensive Care Unit Nurse Physician Questionnaire
Pre: Pre-intervention
Post: Post-intervention
